# Quiet residents of the vaginal epithelium: immunohistochemical study on the distribution and role of human vaginal Merkel cells

**DOI:** 10.1186/s12905-026-04331-3

**Published:** 2026-02-12

**Authors:** Simona Polakovičová, Ivan Varga, Barbora Filová, Luana Sallicandro, Jaroslav Voller, Bernard Fioretti, Alexandra Krištúfková

**Affiliations:** 1 Institute of Histology and Embryology, Faculty of Medicine, Comenius University in Bratislava, Sasinkova Street 4, Bratislava, 811 08 Slovakia; 2 Institute of Anatomy and Anthropology, Rīga Stradiņš University, Riga, LV- 1010 Latvia; 3 Department of Chemistry, Biology and Biotechnology, The Università degli Studi di Perugia, Perugia, 061 23 Italy; 4 Faculty of Healthcare Studies, University of Western Bohemia, Pilsen, 301 00 Czech Republic; 5 First Department of Gynaecology and Obstetrics, Faculty of Medicine, Comenius University and University Hospital, Bratislava, 851 07 Slovakia

**Keywords:** Merkel cell, Human vagina, Immunohistochemistry, Neuroendocrine function

## Abstract

**Background:**

Although Merkel cells (MCs) are well-established mechanoreceptors in human skin, their function within the vaginal epithelium remains undefined. The aim of the present histological study was to investigate whether MCs located in the stratified epithelium of the anterior wall of the human vagina exhibit similar mechanosensory functions to those observed in the skin.

**Methods:**

Immunohistochemical analysis was performed on vaginal wall samples from eight women undergoing transabdominal or laparoscopic surgery. Immunohistochemical markers including cytokeratin 20 (CK20), neuron-specific enolase (NSE), synaptophysin (SYN), chromogranin A (CHRA), vasoactive intestinal peptide (VIP), calcitonin gene-related peptide (CGRP), PIEZO2 and protein gene product 9.5 (PGP9.5) were used to identify MCs and assess their distribution and phenotype. To better demonstrate the connection of MCs with nerve fibres, we used sequential double immune-enzymatic technique with different markers for MCs and for nerve fibres (primary antibodies against CK20 and PGP 9.5) and confirmed that none of examined MCs was in contact with the nerve fibre.

**Results:**

MCs were identified as CK20-positive in all specimens (100%), NSE-positive in 88%, SYN-positive in 25%, CHRA-positive in 100%, and VIP-positive in 25%. No samples demonstrated positivity for CGRP and PIEZO2. MCs were localized predominantly in the basal epithelial layers as solitary cells, with CK20 the most effective detection method. Sequential double immune-enzymatic technique confirmed non- innervated Merkel cells with dendritic morphology.

**Conclusions:**

This is the first study to address the possible function of MCs in the vaginal epithelium. The absence of PIEZO2 and CGRP expression, and low expression of VIP and SYN, suggests a non-mechanosensory and non-nociceptive role for MCs in the vaginal epithelium. The immunophenotypic profile supports a potential endocrine (paracrine or autocrine) function distinct from their role in human skin. Vaginal MCs probably form part of the neuroendocrine system of the vagina and maintain vaginal epithelial homeostasis and regeneration.

## Introduction

**Fig. 1:**
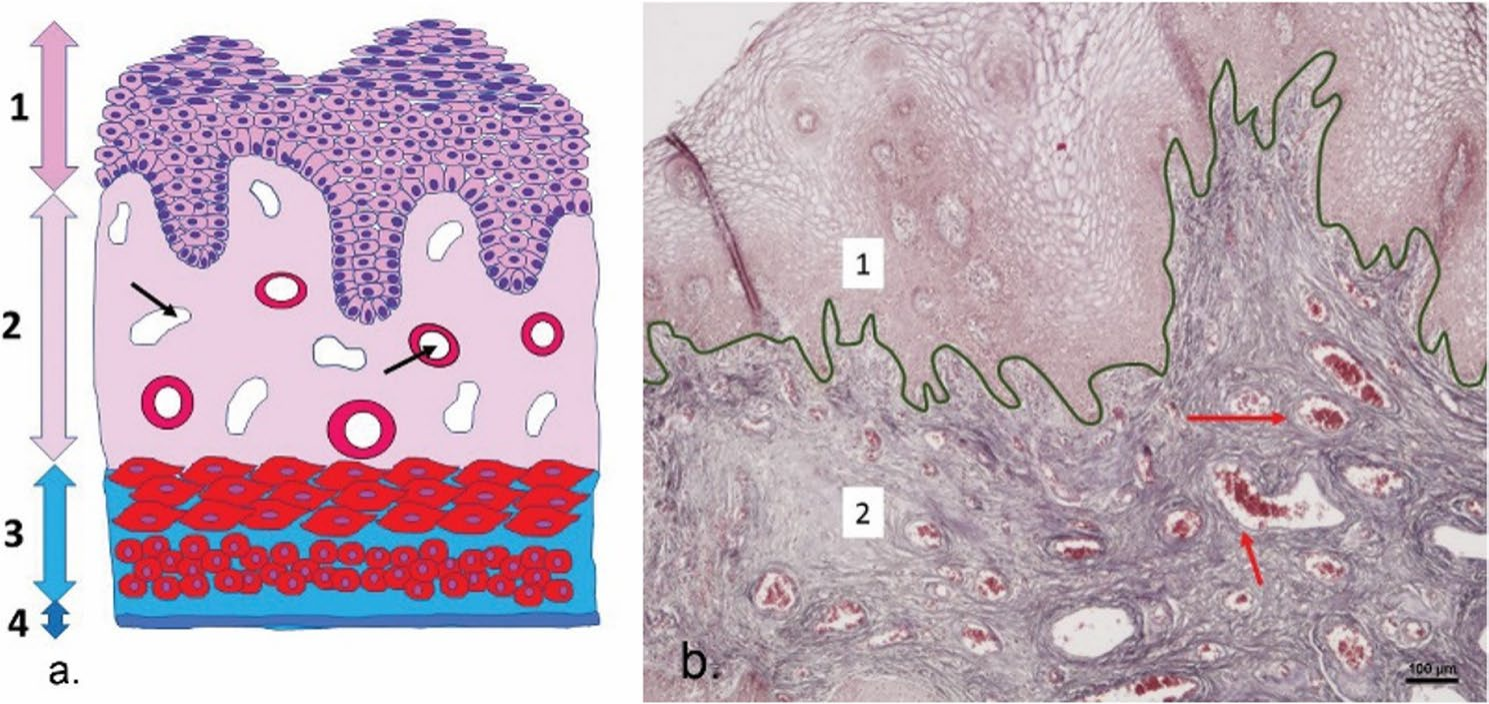
Vaginal wall and mucosa. **a** Schematic of the vaginal wall; **b **Photomicrograph of the vaginal mucosa in Azan staining, Magnification 40×, 1 – stratified squamous non-keratinised epithelium, 2 – connective tissue layer with numerous and dilated venules resembling cavernous / spongy erectile tissue, 3 – muscular layer, 4 – adventitia, arrows - blood vessels, dark green line marks the boundary between epithelium and connective tissue (archive of the Institute of Histology and Embryology, Comenius University in Bratislava, Slovakia)

The human vaginal wall comprises an inner mucosa, a middle muscular layer, and an outermost connective tissue coat called the adventitia (Fig. 1a). Vaginal mucosa is lined by stratified squamous non-keratinised epithelium and a subepithelial connective tissue layer. The deeper areas of the vaginal mucosa contain numerous thin-walled veins (Fig. 1b) resembling cavernous / erectile tissue, called the spongy layer of the vagina [1]. During sexual arousal, vaginal blood flow increases, which enhances vaginal transudation and lubrication; thus facilitating painless penile penetration and creating erectile tissue tumescence [2]. Within the anterior wall of the vagina, periurethral glands are present, recently named as the female prostate, or in the past as Skene’s paraurethral glands [3, 4]. Anatomical studies have suggested that the anterior vaginal wall is more sensitive compared with the posterior vaginal wall and therefore considered that the Gräfenberg spot (hereafter the “G-spot”), as a neurovascular complex, is located in the anterior-distal wall of the vagina [5]. Further studies finding that the clitoris, urethra including the female prostate, the anterior wall of vagina, and surrounding tissues may function together as a whole during female orgasm, and the concept of the clitoral–urethrovaginal complex was proposed to reveal the mechanism of female orgasm [6, 7]. In general, the more distal areas of the vaginal wall have more nerve fibres compared with the more proximal parts. Also, the anterior wall is more densely innervated than the posterior wall [8]. Vaginal stimulation of the anterior vaginal wall leads some women to orgasm due to stimulation of the clitoral–urethrovaginal complex. Thus, the vagina is not a passive organ but a highly dynamic structure with an active role in sexual arousal and intercourse [9].

Human Merkel cells (MCs) were first described by Friedrich S. Merkel in 1875 and named in German as “Tastzellen” (touch cells) assuming a sensory touch function within the skin [10]. MCs are multifunctional intraepithelial cells found in both hairy and glabrous skin, where they function as mechanoreceptors in regions of high tactile sensitivity. They can be defined as epidermal sensory end-organs. Due to their interactions with slowly adapting low-threshold mechanoreceptor afferents neurons, MCs are considered to be part of the main tactile terminal organ involved in the light touch sensation [11]. They are especially numerous in stratified epithelium of the lips, hard palate, palms, finger pads, proximal nail fold, and dorsum of the feet [12]. Within skin, MCs are the most numerous on volar skin, and least on genital skin [13]. Additionally, MCs have also been identified in other epithelia; including those of the gingiva [14], eyelids [15], and oesophagus [16]. Within the female external genitalia, Schober et al. [17] demonstrated their presence in the epithelium of the labia minora and in our previous study we immunohistochemically detected CK20-positive MCs in the human vaginal epithelium [18].

In 1981, Perry and Whipple [19] identified for the first time the G-spot as an area of exceptional sexual sensitivity located in the anterior wall of the vagina. Nevertheless, the existence or non-existence of the G-spot remains a matter of scientific debate and many scientists demonstrated its presence through significantly higher innervation of some parts of the distal anterior vaginal wall than the surrounding areas [20]. Since MCs are connected to nerve fibres within the skin, such that they can provide fine mechanoreception complexes, we assume that vaginal MCs also have a similar mechanoreceptor function. Therefore, our hypothesis is that MCs have peptides and proteins associated with sensation, or maybe endocrine, and immune functions.

## Materials and methods

Tissue samples of the anterior vaginal wall were obtained from eight women undergoing transabdominal or laparoscopic surgery for various indications at the First Department of Gynaecology and Obstetrics, Comenius University and University Hospital in Bratislava, Slovakia. The patients’ mean age was 62.6 ± 12.6 years (range: 42–77 years). The study was conducted in accordance with the Declaration of Helsinki and approved by the local hospital ethics committee (St. Cyril and Methodius Hospital in Bratislava) approved on April 23, 2025. Informed consent was obtained from all subjects involved in the study. Tissue samples were fixed in formalin for 24 h, sectioned longitudinally in accordance with the vaginal length, embedded in paraffin, and cut into 5-µm sections (a standard formalin-fixed paraffin-embedded method). Routine histological processing and haematoxylin and eosin staining were performed. Sections appropriate for immunohistochemical analysis were subsequently cut at 5-µm thickness, deparaffinized, rehydrated, rinsed in distilled water, immersed in antigen retrieval solution (low and high pH) and heated in microwave. For blocking of endogenous peroxidases immersed in 3% hydrogen peroxide at room temperature. For background blocking, we used 1% bovine serum albumin (BSA). Immunohistochemistry employed primary antibodies against the following: cytokeratin 20 (CK20), neuron-specific enolase (NSE), synaptophysin (SYN), chromogranin A (CHRA), vasoactive intestinal peptide (VIP), calcitonin gene-related peptide (CGRP), PIEZO2 and anti-PGP9.5 antigens (more details in Table 1) and stained to detect the expression of antigens typical for MCs, endocrine cells, and / or nerve fibres and nerve endings. Immuno-complexes were visualized with the immunoperoxidase staining technique using dark-brown product (diaminobenzidine as a chromogen), and to better orientation within the tissues, cell nuclei were stained with Mayer’s haematoxylin in dark blue.

**Table 1:** Characteristics of all antibodies used (in alphabetical order)

Antibodies against	Short description with a focus on current research	Company	Host	Isotype	Clonality	Dilution
Calcitonin Gene-Related Peptide (CGRP)	Sensory neuropeptide, present in hair follicle–associated Merkel cells with mechanoreceptor function [21]	Invitrogen	Rabbit	IgG	Polyclonal PA5-114929	1:1000
Chromogranin A (CHRA)	One of most widely used markers for neuroendocrine cells and their tumours [22]	Ventana	Mouse	IgG1, Kappa	Monoclonal (LK2H10) 05267056001	RTU
Cytokeratin 20 (CK20)	Low molecular weight cytokeratin, present in Merkel cells as well as Merkel cell carcinomas [23]	DAKO	Mouse	IgG2, Kappa	Monoclonal (Ks20,8) IS777	RTU
Neuron-Specific Enolase (NSE)	Protein primarily found in neurons, neuroendocrine cells, and neuroendocrine neoplasms [24]	DAKO	Mouse	IgG1, Kappa	Monoclonal (BBS/NC/VI-H14) IR612	RTU
PIEZO2	Transmembrane protein forming part of an ion channel required for mechano-transduction; present as Merkel cells and Merkel cell–neurite complexes as well [25]. Also, Merkel cell carcinoma display immunoreactivity [26]	Novus biologicals	Rabbit	IgG	Polyclonal (NBP1-78538)	1:400
Protein gene product 9.5 (PGP9.5)	Cytoplasmic neuronal and neuroendocrine marker [27]. Widely used to stain neuronal cell bodies and axons in both the central and peripheral nervous systems [28].	Abcam	Rabbit	IgG	Monoclonal (EPR4118)	1:250
Synaptophysin (SYN)	Major integral glycoprotein of the synaptic vesicle membrane in axons and endocrine cells [29], as well as Merkel cell carcinoma [30]	DAKO	Mouse	IgG1, Kappa	Monoclonal (DAK-SYNAP) IR660	RTU
Vasoactive Intestinal Polypeptide (VIP)	Peptide present in dense core granules of dermal Merkel cells [21]	Invitrogen	Rabbit	IgG	Polyclonal PA5-78224	1:1000

*RTU*  Ready to use

During the preparation of material for sequential double immunoenzymatic technique for visualization of Merkel cells and nerve fibres, three serial sections were cut onto a single slide to better capture MCs, because the size of MCs is approximately 10–15 μm and the section thickness was 5 μm. The primary antibodies against CK20 and PGP 9.5 were applied in sequence, detected and visualized individually with two different detection (horseradish peroxidase / Magenta) and (horseradish peroxidase / diaminobenzidine). Primary antibodies were different host (mouse, rabbit). To better orientation within the tissues, cell nuclei were stained with Mayer’s haematoxylin in dark blue. For the positive control, we used samples from the skin of human fingers for CK20, NSE and SYN antibody, large intestine for CHRA antibody, brain tissue for VIP and PGP 9.5 antibodies, spinal cord for CGRP and skin from eyelid for PIEZO2 antibody. These samples were from the archive of the Institute of Histology and Embryology, Comenius University in Bratislava, Slovakia.

These specimens were examined using a Nikon Eclipse 80i light microscope (Tokyo, Japan), and images were acquired with a Nikon DS-Fi1 camera. For each patient, epithelial length was measured under the light microscope, and the number of intraepithelial MCs positive for each marker was counted using a graduated ocular lens to calculate numerical density.

## Results

This study confirmed the presence of CK20-positive MCs in each sample (100%), NSE-positive MCs in 7/8 samples (88%), SYN-positive MCs in 2/8 samples (25%), CHRA-positive MCs in 8/8 samples (100%), and VIP-positive MCs in 2/8 samples (25%). No CGRP and PIEZO2-positive MCs were detected in any of the samples from the anterior vaginal wall (Table 2). Across all samples, MC diameters ranged from 10 to 15 μm. The cells were predominantly localized as solitary cells in the basal regions of the epithelial layer.

**Table 2:** Distribution of immunohistochemical markers in Merkel cells of the human vaginal epithelium

Antibodies against	Positivity of cells in eight samples from anterior vaginal wall
CGRP	0/8 = 0%
Chromogranin A	8/8 = 100%
CK20	8/8 = 100%
NSE	7/8 = 88%
PIEZO2	0/8 = 0%
Synaptophysin	2/8 = 25%
VIP	2/8 = 25%

**Fig. 2:**
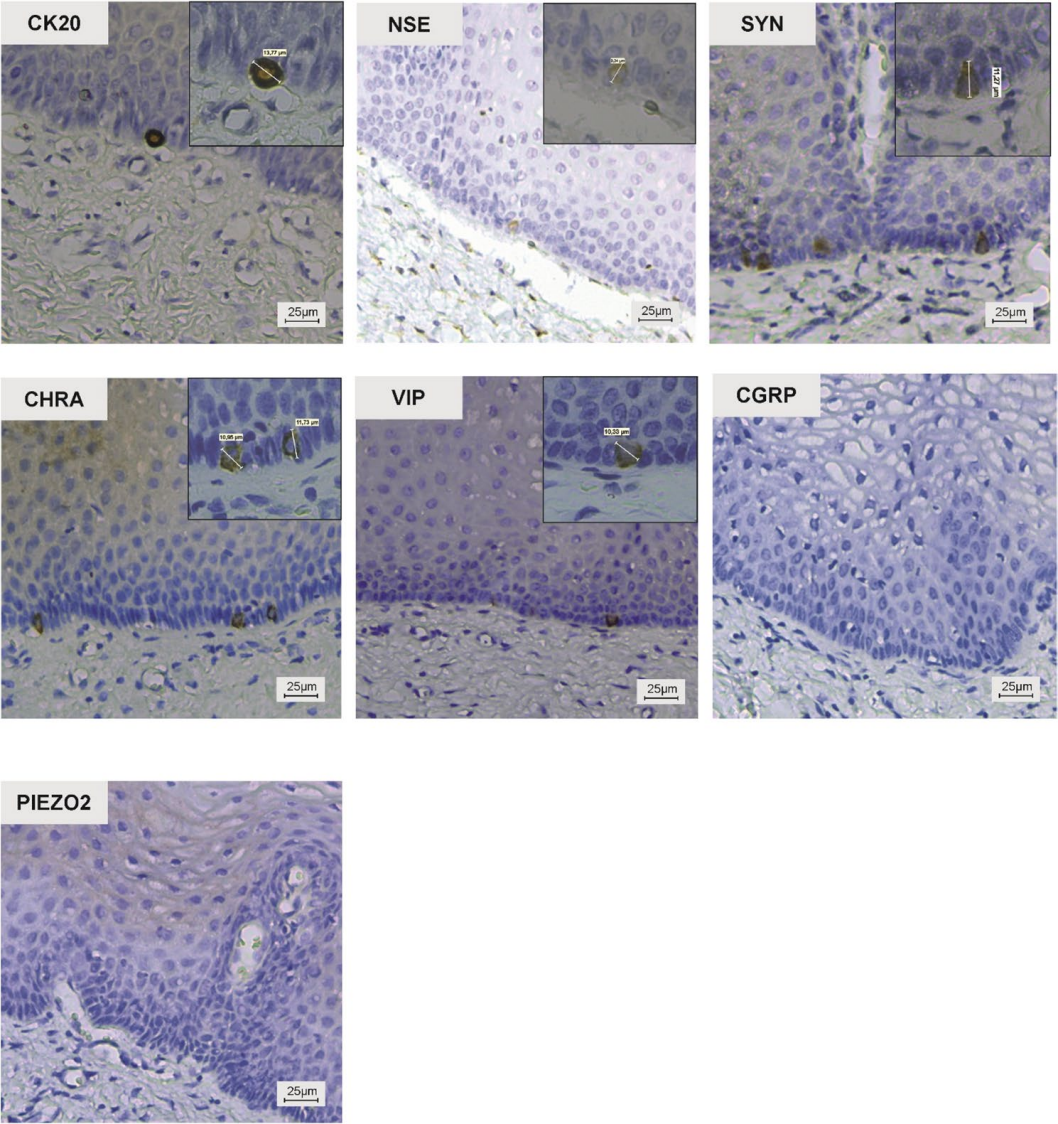
Immunoperoxidase staining. CK20, NSE, SYN, CHRA, VIP positive and CGRP and PIEZO2 negative -Merkel cells in adult human anterior vaginal epithelium. Magnification 200×, insets 400×

CK20, SYN, CHRA, and VIP-positive MCs were easily identified in the basal layer of the stratified epithelium of vagina due to their oval shape and larger diameter compared with surrounding columnar or cuboidal epithelial cells (Fig. 2). CK20-positive MCs displayed a characteristic peripheral ring-like halo and demonstrated the strongest and most consistent immunoreactivity among all markers examined. SYN, CHRA, and VIP-positive MCs were also distinguishable by their oval shape and, in some cases, short cytoplasmic processes. These cells exhibited a granular cytoplasmic staining pattern, with immunopositivity present in both the cell body and cytoplasmic processes (Fig. 2). NSE-positive MCs were more difficult to identify in many specimens; however, their immunoreactivity was sufficient for detection and displayed a very fine granular distribution.

Quantitative analysis (Table 3) indicated that CK20-positive MCs were present in all eight tissue samples, ranging from 3 to 15 cells per sample, with a mean density of 7 cells per 1.0 cm of epithelium. NSE-positive MCs were detected in seven of the eight samples, with counts ranging from 1 to 12 cells. One specimen had the highest count of 12 NSE-positive MCs, whereas another contained only a single cell; one sample lacked NSE immunoreactivity entirely. SYN-positive MCs were the least frequent, present in only two samples: one with 16 cells and another with 4, whereas the remaining six samples were negative for SYN. CHGA-positive MCs were identified in all samples, ranging from 1 to 17 cells per sample. VIP-positive MCs were detected in two samples, ranging from 1 to 2 cells. No CGRP- or PIEZO2-positive MCs were identified in any of the eight specimens.

**Table 3:** Quantitative distribution of marker-positive Merkel cells in the vaginal epithelium

Patient age (years)	CK20 + cells	NSE+ cells	SYN+ cells	CHRA+ cells	PIEZO+ cells	CGRP+ cells	VIP+ cells
42	6	9	0	15	0	0	1
50	3	1	0	17	0	0	0
59	6	10	0	2	0	0	0
61	15	0	16	14	0	0	0
66	10	4	4	15	0	0	2
70	6	3	0	2	0	0	0
76	4	2	0	1	0	0	0
77	5	12	0	8	0	0	0

**Fig. 3:**
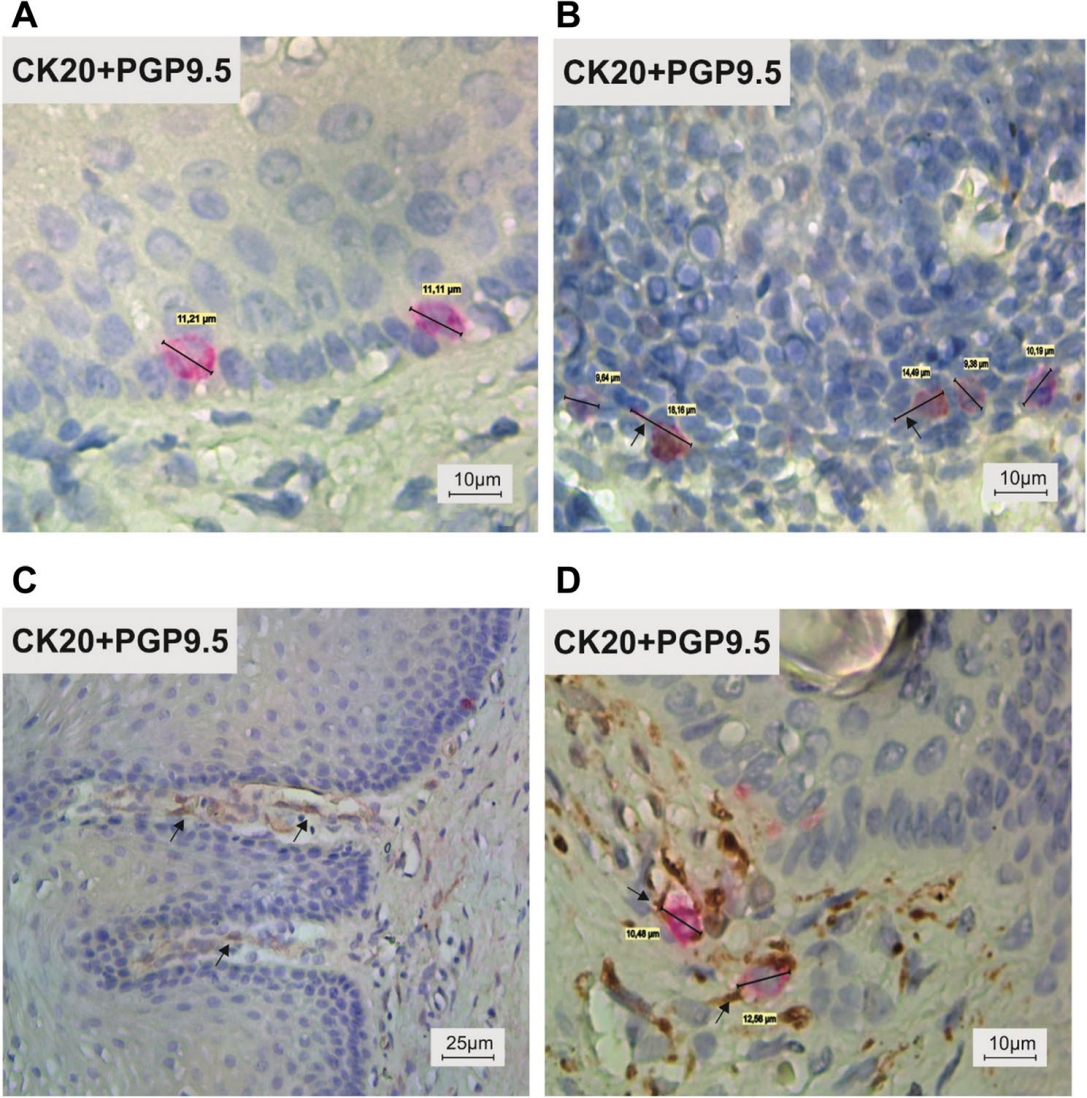
**S**equential double immunoenzymatic technique for visualization of Merkel cells and nerve fibers in serial sections of adult human anterior vaginal epithelium **A**, **B**, **C**. Vaginal Merkel cells are characterized by the presence of cytoplasmic projections (Fig. 3B – arrows) and a dendritic morphology. In vaginal wall, thin nerve fibers are located in the connective tisuue of lamina propria (Fig. 3C - arrows). Figure 3D shows the positive control consisting of human eyelid skin, where nerve fibers are in direct contact with Merkel cells (Fig. 3D – arrows). Magnification **A**, **B**, and **D** 630×, **C** 200×

To better demonstrate the connection of MCs with nerve fibers, we used double labelling with two different markers: for MCs and for nerve fibers and confirmed that none of examined MCs was in contact with the nerve fiber. CK20 positive MCs were easily identified in the basal layer of the stratified epithelium. Some of the MCs were oval some were round in shape and larger diameter (10–15 μm) compared with surrounding columnar or cuboidal epithelial cells (Fig. 3A). Some MCs had very thin cytoplasmatic processes and had dendritic shape (Fig. 3B). PGP 9.5 positive thin nerve fibers were located in the connective tissue beneath surface epithelium, but non-were in close contact with MCs (Fig. 3C). As comparison with the outer hair root sheath from the human eyelid (as positive control) CK 20- positive MCs were oval in shape measured 10–13 μm. They were in close contact with nerve fibers (Fig. 3C).

## Discussion

Merkel cells represent a minor population of total epidermal cells; on average, 1.5% of the epidermal cell population in samples isolated from glabrous skin and 1.6% from hairy skin [31]. They are usually scattered along the dermo–epidermal junction of the skin. MCs and MC interactions with afferent neurons (so-called MC complexes) form complex and rare epidermal sensory end-organs. Yet MCs are neuroendocrine cells that are ultrastructurally distinguishable from other epidermal cell types by the presence of electron-dense neurosecretory granules opposite the nerve endings located in the dermis [11]. The aim of the present histological study was to investigate whether MCs located in the stratified epithelium of the anterior wall of the human vagina exhibit similar mechanosensory functions to those observed in the skin. The innervation of this genital region remains poorly understood, and existing literature lacks clarity and detail. If vaginal MCs are fine mechanoreceptors, they could be involved in formation of the G-spot. To determine the immunoprofile of MCs in this previously unexplored anatomical location and to classify them functionally, we employed seven antigenic markers, which are typical for skin MCs: CK20, NSE, SYN, CHRA, VIP, CGRP, and PIEZO2.

Intraepidermal MCs, MC hyperplasia, MC carcinoma, and MC carcinoma metastases in sentinel lymph nodes can all be confirmed by immunohistochemistry using CK20 staining [32–34]. Vaginal MCs also exhibited 100% cytoplasmic positivity for CK20, with an average number of seven MCs per centimetre of vaginal epithelium. MCs were located solitary in the basal layer of the vaginal epithelium, near the basement membrane.

Touch is an essential way for humans to sense the physical world. The vaginal epithelium is responsive to mechanical and thermal stimuli, including pressure, stretch, pain, and temperature [35]. The PIEZO2 and synaptophysin markers were used to confirm or exclude mechanoreceptive functionality of vaginal MCs, whereas CGRP and VIP were used to assess potential nociceptive properties (modulate sensory processing of touch). PIEZO2 is characteristically expressed by skin MCs [26, 36]. Skin MCs convert mechanical stimuli into release of neurotransmitters with the participation of PIEZO2 channels, membrane potentials, and Ca^2+^ transients. When skin MCs are compressed, the opening of PIEZO2 channels leads to depolarization of membrane potentials and influx of Ca^2+^. Therefore, PIEZO2 channels excite skin MCs in a manner that produces sustained neurotransmitter release [37]. SYN can be detected in MCs and additionally, full co-localization between SYN and PIEZO2 is present in some skin MCs [29]. VIP and CGRP in granules of epidermal MCs may function as a neuromodulator that influences the activity of the sensory nerve terminal supplying the cell [38]. However, we were surprised that vaginal MCs were positive for neither PIEZO2 nor CGRP. Furthermore, only in two samples from eight (25%); and even then, only rarely did we find VIP-positive vaginal MCs. In two samples from eight (25%), MCs were positive for SYN. This suggests that vaginal MCs probably have neither a mechanoreceptor nor nociceptive function, and thus differ from MCs of the skin. This finding supports the concept of Merkel cell morphological and functional heterogeneity proposed by Nakafusa et al. [39] or Michalak -Micka et al. [40], according to some of Merkel cells are connected to nerve fibers and others are not.

Vaginal MCs exhibit positivity for two typical markers of endocrine cells: chromogranin A and NSE. NSE is a specific marker for human skin MCs [41] and MC carcinomas [42]. Similarly, CHRA is typically present in human skin MCs [43]. Although most MCs of the skin are connected to sensory neurons, a subset of MCs is not innervated, particularly in the palatine mucosa of rodents. Many of these cells have a dendritic shape and accumulate neurosecretory granules. The function of non-innervated skin MCs remains unknown [44]. Similarly, the function of non-innervated vaginal MCs is unknown. Based on the positivity of CHRA and NSE, we suggest their secretory (paracrine or endocrine) function. One of the possible paracrine functions of MCs may be the regulation of epithelial regeneration and wound healing. In animal models, specifically in full-thickness wounds of the inferior labial mucous membrane in rabbits, an increase in the number of MCs was observed during the healing process [45]. Similarly, within the hair follicles of rats, repeated shaving leads to an increased number of MCs, suggesting that MCs may also play a role in stimulating hair regeneration [46]. The role of non-innervated MCs in the homeostasis of stratified epithelia in various anatomical regions of the body, as well as their role in epithelial regeneration, represents one of the possible directions for further research.

Vaginal MCs may be a source of malignancies, even though vaginal MC carcinomas are extremely rare. Albores-Saavedra et al. [47] analysed 3870 cases of MC carcinomas from 1973 to 2006 and only one case was localised in the vagina. Based on the immunophenotype of vaginal MCs, these may play role in the development of some primary neuroendocrine carcinomas of the vagina, which are rare among female genital tract tumours. These vaginal neuroendocrine carcinomas with MC carcinoma phenotype are cytokeratin 20, NSE, and chromogranin A positive [48].

A limitation of this study is a higher, mostly post-menopausal age of women (mean age 62.6 ± 12.6 years). The vaginal epithelium is highly hormone-responsive. Reduced oestrogen level lead to a decrease in epithelial layers, vascularization, and sensory-motor innervation [49]. However, it is not known how menopause and changes in sex hormone levels affect the number and function of individual vaginal epithelial cell types. We identified also another limitation. Although axonal markers such as NSE were used, identifying the nerve fiber supplying a MC can be challenging and may require more sensitive techniques, such as confocal immunofluorescence, as were demonstrated by García-Mesa et al. [25]. While this study did not document nerve fibers associated with Merkel cells, their detection may require more detailed analyses and higher-sensitivity immunohistochemical methods.

## Conclusions

Our findings suggest the presence of non-innervated MCs in the human vaginal epithelium that lack mechanosensory and nociceptive capabilities. The immunoprofile of MCs in the human vagina supports the hypothesis that these cells may exert endocrine (paracrine or autocrine) functions that are distinct from their known typical cutaneous role. Vaginal MCs probably form part of the neuroendocrine system of the vagina and maintain vaginal epithelial homeostasis and regeneration. Additionally, vaginal MCs can be a source for some rare neuroendocrine carcinomas of the vagina.

## Data Availability

The dataset used and/or analyzed during the current study are available from the corresponding author on reasonable request.

## Abbreviations

CGRP: Calcitonin Gene–Related Peptide
CHRA: Chromogranin A
CK20: Cytokeratin 20
MC: Merkel Cell
NSE: Neuron–Specific Enolase
PGP 9.5: protein gene product 9.5
RTU: Ready To Use
SYN: Synaptophysin
VIP: Vasoactive Intestinal Polypeptide

## References

[1] Varga I, Hammer N, Pavlíková L, Poilliot A, Klein M, Mikušová R. Terminological discrepancies and novelties in the histological description of the female genital system: proposed amendments for clinical-translational anatomy. Anat Sci Int. 2024;99(4):469–80. 10.1007/s12565-024-00772-8. 38683308 10.1007/s12565-024-00772-8 PMC11303487

[2] Levin RJ. Recreation and procreation: a critical view of sex in the human female. Clin Anat. 2015;28(3):339–54. 10.1002/ca.22495. 25511503 10.1002/ca.22495

[3] Zaviacic M, Jakubovská V, Belosovic M, Breza J. Ultrastructure of the normal adult human female prostate gland (Skene’s gland). Anat Embryol (Berl). 2000;201(1):51–61. 10.1007/pl00022920. 10603093 10.1007/pl00022920

[4] Kotil G, Barut C. A vulvar anatomy description comparison between the anatomy and gynecological textbooks. Bratisl Med J. 2025;126:1362–70. 10.1007/s44411-025-00229-y.

[5] Ostrzenski A. G-Spot anatomy and its clinical significance: A systematic review. Clin Anat. 2019;32(8):1094–101. 10.1002/ca.23457. 31464000 10.1002/ca.23457

[6] Wei L, Jiang H, Jiang T. The relationship between clitourethrovaginal complex and female orgasm. Arch Gynecol Obstet. 2023;308(6):1697–702. 10.1007/s00404-023-06977-y. 36854986 10.1007/s00404-023-06977-y

[7] Jannini EA, Buisson O, Rubio-Casillas A. Beyond the G-spot: clitourethrovaginal complex anatomy in female orgasm. Nat Rev Urol. 2014;11(9):531–8. 10.1038/nrurol.2014.193. 25112854 10.1038/nrurol.2014.193

[8] Hilliges M, Falconer C, Ekman-Ordeberg G, Johansson O. Innervation of the human vaginal mucosa as revealed by PGP 9.5 immunohistochemistry. Acta Anat (Basel). 1995;153(2):119–26. 10.1159/000147722. 8560964 10.1159/000147722

[9] Arias-Castillo L, García L, García-Perdomo HA. The complexity of female orgasm and ejaculation. Arch Gynecol Obstet. 2023;308(2):427–34. 10.1007/s00404-022-06810-y. 36208324 10.1007/s00404-022-06810-y

[10] Moll I, Roessler M, Brandner JM, Eispert AC, Houdek P, Moll R Human Merkel cells–aspects of cell biology, distribution and functions. Eur J Cell Biol. 2005;84(2–3):259–71. 10.1016/j.ejcb.2004.12.023. 15819406 10.1016/j.ejcb.2004.12.023

[11] Bataille A, Le Gall C, Misery L, Talagas M. Merkel cells are multimodal sensory cells: a review of study methods. Cells. 2022;11(23):3827. 10.3390/cells11233827. 36497085 10.3390/cells11233827 PMC9737130

[12] Lacour JP, Dubois D, Pisani A, Ortonne JP. Anatomical mapping of Merkel cells in normal human adult epidermis. Br J Dermatol. 1991;125(6):535–42. 10.1111/j.1365-2133.1991.tb14790.x. 1722110 10.1111/j.1365-2133.1991.tb14790.x

[13] Boot PM, Rowden G, Walsh N. The distribution of Merkel cells in human fetal and adult skin. Am J Dermatopathol. 1992;14(5):391–6. 10.1097/00000372-199210000-00003. 1415956 10.1097/00000372-199210000-00003

[14] Straka M, Polák Š, Straková Trapezanlidis M, Varga I. What we know about the cellular microenvironment of clinically healthy human gingiva? An immunohistochemical and histological study. Biologia. 2017;72(1):105–11. 10.1515/biolog-2017-0011.

[15] May CA, Osterland I. Merkel cell distribution in the human eyelid. Eur J Histochem. 2013;57(4):e33. 10.4081/ejh.2013.e33. 24441186 10.4081/ejh.2013.e33 PMC3896035

[16] Polakovičová S, Mikušová R, Polák Š. Merkel cells in the stratified squamous nonkeratinized epithelium of the human oesophagus. Biologia. 2013;68(4):743–6.

[17] Schober JM, Martín-Alguacil N, Cooper RS, Aardsma N, Mayoglou L, Litvin Y, Pfaff D. Identification of Merkel cells in the labia minora skin of prepubertal girls. J Genit Syst Disor. 2016;5:2. 10.4172/2325-9728.1000154.

[18] Polakovičová S, Csöbönyeiová M, Filova B, Borovský M, Maršík L, Kvasilová A, Polák Š. Merkel-like cell distribution in the epithelium of the human vagina. An immunohistochemical and TEM study. Eur J Histochem. 2018;62(1):2836. 10.4081/ejh.2018.2836. 29569875 10.4081/ejh.2018.2836 PMC5827109

[19] Perry JD, Whipple B. Pelvic muscle strength of female ejaculators. Evidence in support of a new theory of orgasm. J Sex Res. 1981;17:22–39. 10.1080/00224498109551095.

[20] Song YB, Hwang K, Kim DJ, Han SH. Innervation of vagina: microdissection and immunohistochemical study. J Sex Marital Ther. 2009;35(2):144–53. 10.1080/00926230802716195. 19266382 10.1080/00926230802716195

[21] Tachibana T, Nawa T. Immunohistochemical reactions of receptors to met-enkephalin, VIP, substance P, and CGRP located on Merkel cells in the rat sinus hair follicle. Arch Histol Cytol. 2005;68(5):383–91. 10.1679/aohc.68.383. 16505584 10.1679/aohc.68.383

[22] Tomita T. Significance of chromogranin A and synaptophysin in pancreatic neuroendocrine tumors. Bosn J Basic Med Sci. 2020;20(3):336–46. 10.17305/bjbms.2020.4632. 32020844 10.17305/bjbms.2020.4632 PMC7416176

[23] Rabban JT, Soslow RA, Zaloudek CZ. Chapter 18 - Immunohistology of the female genital tract. In: Dabbs DJ, Ed, editors. Diagnostic immunohistochemistry (Third Edition). W.B. Saunders; 2010. p.690–762. 10.1016/B978-1-4160-5766-6.00022-4. ISBN 9781416057666.

[24] Mjønes P, Sagatun L, Nordrum IS, Waldum HL. Neuron-specific enolase as an immunohistochemical marker is better than its reputation. J Histochem Cytochem. 2017;65(12):687–703. 10.1369/0022155417733676. 28972818 10.1369/0022155417733676 PMC5714096

[25] García-Mesa Y, Feito J, Cuendias P, García-Piqueras J, Germanà A, García-Suárez O, Martín-Biedma B, Vega JA. The acquisition of mechanoreceptive competence by human digital Merkel cells and sensory corpuscles during development: an immunohistochemical study of PIEZO2. Ann Anat. 2022;243:151953. 10.1016/j.aanat.2022.151953. 35523396 10.1016/j.aanat.2022.151953

[26] García-Mesa Y, Martín-Sanz R, García-Piqueras J, Cobo R, Muñoz-Bravo S, García-Suárez O, Martín-Biedma B, Vega JA, Feito J. Merkel cell carcinoma display PIEZO2 immunoreactivity. J Pers Med. 2022;12(6):894. 10.3390/jpm12060894. 35743679 10.3390/jpm12060894 PMC9224776

[27] Ramieri G, Panzica GC, Viglietti-Panzica C, Modica R, Springall DR, Polak JM. Non-innervated Merkel cells and Merkel-neurite complexes in human oral mucosa revealed using antiserum to protein gene product 9.5. Arch Oral Biol. 1992;37(4):263–9. 10.1016/0003-9969(92)90048-D. 1387783 10.1016/0003-9969(92)90048-d

[28] Day IN, Thompson RJ. UCHL1 (PGP 9.5): neuronal biomarker and ubiquitin system protein. Prog Neurobiol. 2010;90(3):327–62. 10.1016/j.pneurobio.2009.10.020. 19879917 10.1016/j.pneurobio.2009.10.020

[29] García-Mesa Y, García-Piqueras J, Cuendias P, Cobo R, Martín-Cruces J, Feito J, García-Suarez O, Biedma BM, Vega JA. Synaptophysin is a selective marker for axons in human cutaneous end organ complexes. Ann Anat. 2022;243:151955. 10.1016/j.aanat.2022.151955. 35588932 10.1016/j.aanat.2022.151955

[30] Hou X, Lv Q, Lv Z. Breast skin Merkel cell carcinoma: a case report. Am J Transl Res. 2025;17(5):3554–9. 10.62347/YFTJ3672. 40535693 10.62347/YFTJ3672 PMC12170398

[31] Fradette J, Larouche D, Fugère C, Guignard R, Beauparlant A, Couture V, Caouette-Laberge L, Roy A, Germain L. Normal human Merkel cells are present in epidermal cell populations isolated and cultured from glabrous and hairy skin sites. J Invest Dermatol. 2003;120(2):313–7. 10.1046/j.1523-1747.2003.12024.x. 12542538 10.1046/j.1523-1747.2003.12024.x

[32] Valiga A, Tababa EJ, Chung HJ, Cha J. Merkel cell hyperplasia versus intraepidermal Merkel cell carcinoma: a comparative study of 2 cases. Am J Dermatopathol. 2023;45(7):505–8. 10.1097/DAD.0000000000002457. 37249368 10.1097/DAD.0000000000002457

[33] Do J, Wang Y, Aung PP, Nagarajan P, Ning J, Curry JL, Ivan D, Lenskaya V, Torres-Cabala CA, Prieto VG, Cho WC. INSM1: A highly sensitive marker for primary and metastatic Merkel cell carcinoma, superior to SOX11, pancytokeratin, and CK20. Hum Pathol. 2025;105838. 10.1016/j.humpath.2025.105838. 10.1016/j.humpath.2025.105838 40505699

[34] Gauci ML, Aristei C, Becker JC, Blom A, Bataille V, Dreno B, Del Marmol V, Forsea AM, Fargnoli MC, Grob JJ, Gomes F, Hauschild A, Hoeller C, Harwood C, Kelleners-Smeets N, Kaufmann R, Lallas A, Malvehy J, Moreno-Ramirez D, Peris K, Pellacani G, Saiag P, Stratigos AJ, Vieira R, Zalaudek I, van Akkooi ACJ, Lorigan P, Garbe C, Lebbé C. European dermatology forum (EDF), the European association of Dermato-Oncology (EADO) and the European organization for research and treatment of cancer (EORTC). Diagnosis and treatment of Merkel cell carcinoma: European consensus-based interdisciplinary guideline - Update 2022. Eur J Cancer. 2022;171:203–31. 10.1016/j.ejca.2022.03.043. 35732101 10.1016/j.ejca.2022.03.043

[35] Vardi Y, Gruenwald I, Sprecher E, Gertman I, Yartnitsky D. Normative values for female genital sensation. Urology. 2000;56(6):1035–40. 10.1016/s0090-4295(00)00850-5. 11113756 10.1016/s0090-4295(00)00850-5

[36] Bataille-Savattier A, Le Gall-Ianotto C, Lebonvallet N, Misery L, Talagas M. Do Merkel complexes initiate mechanical itch? Exp Dermatol. 2023;32(2):226–34. 10.1111/exd.14685. 36208286 10.1111/exd.14685

[37] Mao F, Yang W. How Merkel cells transduce mechanical stimuli: a biophysical model of Merkel cells. PLoS Comput Biol. 2023;19(12):e1011720. 10.1371/journal.pcbi.1011720. 38117763 10.1371/journal.pcbi.1011720 PMC10732429

[38] Cheng-Chew SB, Leung PY. Localisation of VIP-and CGRP-like substances in the skin and sinus hair follicles of various mammalian species. Histochem Cell Biol. 1996;105(6):443–52. 10.1007/BF01457657. 8791103 10.1007/BF01457657

[39] Nakafusa J, Narisawa Y, Shinogi T, Taira K, Tanaka T, Inouge T, Misago N. Changes in the number of Merkel cells with the hair cycle in hair discs on rat back skin. Br J Dermatol. 2006;155(5):883–9. 10.1111/j.1365-2133.2006.07441.x. 17034514 10.1111/j.1365-2133.2006.07441.x

[40] Michalak-Micka K, Rütsche D, Mazzone L, Büchler VL, Moehrlen U, Klar AS, Biedermann T. Human fetal skin derived Merkel cells display distinctive characteristics in vitro and in bio-engineered skin substitutes in vivo. Front Bioeng Biotechnol. 2022;5:10: 983870. 10.3389/fbioe.2022.983870. 10.3389/fbioe.2022.983870 PMC9520781 36185452

[41] Masuda T, Ikeda S, Tajima K, Kawamura T. Neuron-specific enolase (NSE): a specific marker for Merkel cells in human epidermis. J Dermatol. 1986;13(1):67–9. 10.1111/j.1346-8138.1986.tb02903.x. 3522698 10.1111/j.1346-8138.1986.tb02903.x

[42] Gu J, Polak JM, Van Noorden S, Pearse AG, Marangos PJ, Azzopardi JG. Immunostaining of neuron-specific enolase as a diagnostic tool for Merkel cell tumors. Cancer. 1983;52(6):1039–43. 6349776 10.1002/1097-0142(19830915)52:6<1039::aid-cncr2820520619>3.0.co;2-o

[43] Hartschuh W, Weihe E, Yanaihara N. Immunohistochemical analysis of chromogranin A and multiple peptides in the mammalian Merkel cell: further evidence for its paraneuronal function? Arch Histol Cytol. 1989;52(Suppl):423–31. 10.1679/aohc.52.suppl_423. 2510797 10.1679/aohc.52.suppl_423

[44] Boulais N, Misery L. Merkel cells. J Am Acad Dermatol. 2007;57(1):147–65. 10.1016/j.jaad.2007.02.009. 17412453 10.1016/j.jaad.2007.02.009

[45] Tachibana T, Ishizeki K. Merkel cell development in the wound healing in the labial mucosa of adult rabbits. Arch Histol Jap. 1981;44(2):151–65. 10.1679/aohc1950.44.151. 7316693 10.1679/aohc1950.44.151

[46] Wright MC, Logan GJ, Bolock AM, Kubicki AC, Hemphill JA, Sanders TA, Maricich SM. Merkel cells are long-lived cells whose production is stimulated by skin injury. Dev Biol. 2017;422(1):4–13. 10.1016/j.ydbio.2016.12.020. 27998808 10.1016/j.ydbio.2016.12.020 PMC5253117

[47] Albores-Saavedra J, Batich K, Chable-Montero F, Sagy N, Schwartz AM, Henson DE. Merkel cell carcinoma demographics, morphology, and survival based on 3870 cases: a population based study. J Cutan Pathol. 2010;37(1):20–7. 10.1111/j.1600-0560.2009.01370.x. 19638070 10.1111/j.1600-0560.2009.01370.x

[48] Coleman NM, Smith-Zagone MJ, Tanyi J, Anderson ML, Coleman RL, Dyson SW, Reed JA. Primary neuroendocrine carcinoma of the vagina with Merkel cell carcinoma phenotype. Am J Surg Pathol. 2006;30(3):405–10. 10.1097/01.pas.0000194737.95421.9d. 16538063 10.1097/01.pas.0000194737.95421.9d

[49] da Silva Lara LA, da Silva AR, Rosa-E-Silva JC, Chaud F, Silva-de-Sá MF, Meireles E, de Silva AR. Sá Rosa-E-Silva AC. Menopause leading to increased vaginal wall thickness in women with genital prolapse: impact on sexual response. J Sex Med. 2009;6(11):3097–110. 10.1111/j.1743-6109.2009.01407.x. 19656272 10.1111/j.1743-6109.2009.01407.x

